# Survival and stability of strategic mini-implants with immediate or delayed loading under removable partial dentures: a 3-year randomized controlled clinical trial

**DOI:** 10.1007/s00784-022-04805-2

**Published:** 2022-12-06

**Authors:** Torsten Mundt, Friedhelm Heinemann, Janine Müller, Christian Schwahn, Ahmad Al Jaghsi

**Affiliations:** 1 Department of Prosthodontics, Gerodontology and Dental Materials, University Medicine of Greifswald, Walther-Rathenau-Str. 42a, 17475 Greifswald, Germany; 2 Private Practice, Morsbach-Lichtenberg, Germany; 3 College of Dentistry, Clinical Sciences Department, Ajman University, Ajman, United Arab Emirates; 4 Center of Medical and Bio-Allied Health Sciences Research, Ajman University, Ajman, United Arab Emirates

**Keywords:** Denture, Partial, Removable, Randomized controlled trial, Immediate dental implant loading, Mini-implant, Survival rate, Resonance frequency analysis

## Abstract

**Objectives:**

Stability values of mini-implants (MIs) are ambiguous. Survival data for MIs as supplementary abutments in reduced dentitions are not available. The aim of this explorative research was to estimate the 3-year stability and survival of strategic MIs after immediate and delayed loading by existing removable partial dentures (RPDs).

**Material and methods:**

In a university and three dental practices, patients with unfavorable tooth distributions received supplementary MIs with diameters of 1.8, 2.1, and 2.4 mm. The participants were randomly allocated to group A (if the insertion torque ≥ 35 Ncm: immediate loading by housings; otherwise, immediate loading by RPD soft relining was performed) or delayed loading group B. Periotest values (PTVs) and resonance frequency analysis (RFA) values were longitudinally compared using mixed models.

**Results:**

A total of 112 maxillary and 120 mandibular MIs were placed under 79 RPDs (31 maxillae). The 1st and 3rd quartile of the PTVs ranged between 1.7 and 7.8, and the RFA values ranged between 30 and 46 with nonrelevant group differences. The 3-year survival rates were 92% in group A versus 95% in group B and 99% in the mandible (one failure) versus 87% in the maxilla (eleven failures among four participants).

**Conclusions:**

Within the limitations of explorative analyses, there were no relevant differences between immediate and delayed loading regarding survival or stability of strategic MIs.

**Clinical relevance:**

The stability values for MIs are lower than for conventional implants. The MI failure rate in the maxilla is higher than in the mandible with cluster failure participants.

**Clinical trial registration:**

German Clinical Trials Register (Deutsches Register Klinischer Studien, DRKS-ID: DRKS00007589, www.germanctr.de), January 15, 2015.

## Introduction

In jaws with few remaining teeth or unfavorable distribution between quadrants, strategic dental implants can serve as supplementary abutments for a symmetric support and more stability of removable partial dentures (RPD) [1–5]. Posterior-placed implants are also used to support distal extension RPDs [6–9]. Implants can be placed either before the fabrication of the new prosthesis [1, 2] or under an existing RPD [3, 5–9]. Latter studies showed improvements of the RPD function, i.e., chewing efficiency [3, 6, 9], and of patient-immanent factors such as satisfaction with the denture [3, 7, 8], quality of life [5], or nutrient intake [6]. The connection of teeth and implants is still considered as controversial due to the different movement behaviors of natural and artificial abutments, particularly under fixed prostheses [4]. However, this combination is a reliable and predictable treatment modality in RPDs even when different attachments, e.g., double crowns or clasps on teeth and ball attachment on implants, are used [4, 8, 10]. A systematic literature review of combined teeth and implant-retained RPDs revealed implant survival rates between 91.7 and 100% after 2 to 10 years of observation [10]. In a meta-analysis of studies with double crown-retained RPDs supported by implants and teeth, the overall survival rate of implants was 98.7% (95% confidence interval [95% CI]: 97–99.8%) after at least 3 years [11].

Unfortunately, conventional two-piece implants are cost-intensive and sometimes require bone augmentation procedures in narrow alveolar ridges. One-piece mini-implants (MIs) with diameters of 1.8 to 2.9 mm are mainly indicated in atrophied ridges with insufficient width to stabilize removable dentures. Thus, MIs may reduce the invasiveness of the surgical intervention with subsequently less postoperative morbidity and the primary treatment cost [12]. Therefore, MIs are especially suitable for medically compromised and/or financially restricted patients [12–15]. Hereby, especially elderly patients who refuse complex interventions could be encouraged to choose implant-supported rehabilitations [13].

Because of the one-piece design, a total no-load secondary osseointegration is not possible in the edentulous jaws as their main indication. However, this design should prevent microbiological leakage into the surrounding implant tissue [16, 17].

Primary stability seems to be a key factor for successful osseointegration, especially for immediately loaded implants [18]. Therefore, the assessment of MI stability could be a supplementary tool to other clinical or radiographic examination to monitor successful primary and secondary osseointegration [19]. Previous stability data of mandibular MIs using the Periotest device were inconsistent. The mean Periotest values (PVTs) were either in the range of two-piece standard-diameter implants, i.e., < 0 [20, 21], or ranged between + 2 and + 7 [22, 23].

According to some reviews, MI-supported overdentures are a viable and safe treatment option for edentulous mandibular arches with mid-term survival rates > 90%, which is similar to standard-diameter implants [12, 13, 24, 25]. However, the results for immediate loaded MIs to support maxillary overdentures are unfavorable, with survival rates below 80% [13, 14]. Another progressive loading concept involves an initial soft relining of the dentures for 3 to 6 months followed by the connection with the matrices [15, 26]. The survival rates of maxillary MIs were 82.3% after 3 years in a prospective study [26] and 94.3% after 4 years in a retrospective analysis [15]. Prospective studies of strategic MIs under RPDs are extremely rare [13, 27]. There are some short-time observations of MIs for the anterior fixation of free-end RPDs [28, 29].

Meanwhile, a 3-year randomized clinical trial (RCT) was completed, comparing immediate with delayed loading of strategic MIs under existing RPDs with unfavorable tooth distribution [30]. The primary outcome was the radiological bone level change at MIs. First evaluations found marked improvements in the chewing efficiency [31] and patient satisfaction with their RPDs [32]. The improvements occurred faster after immediate loading than delayed MI loading.

The aims of the present explorative analyses were (1) to describe the longitudinal stability values for MIs under different loading conditions, (2) to estimate the MI survival rates, and (3) to evaluate whether PTVs can predict MI losses. We hypothesized less initial MI stability and more MI failures in the immediate than in the delayed loading group as in the study protocol [30].

## Material and methods

### Study procedures

**Fig. 1:**
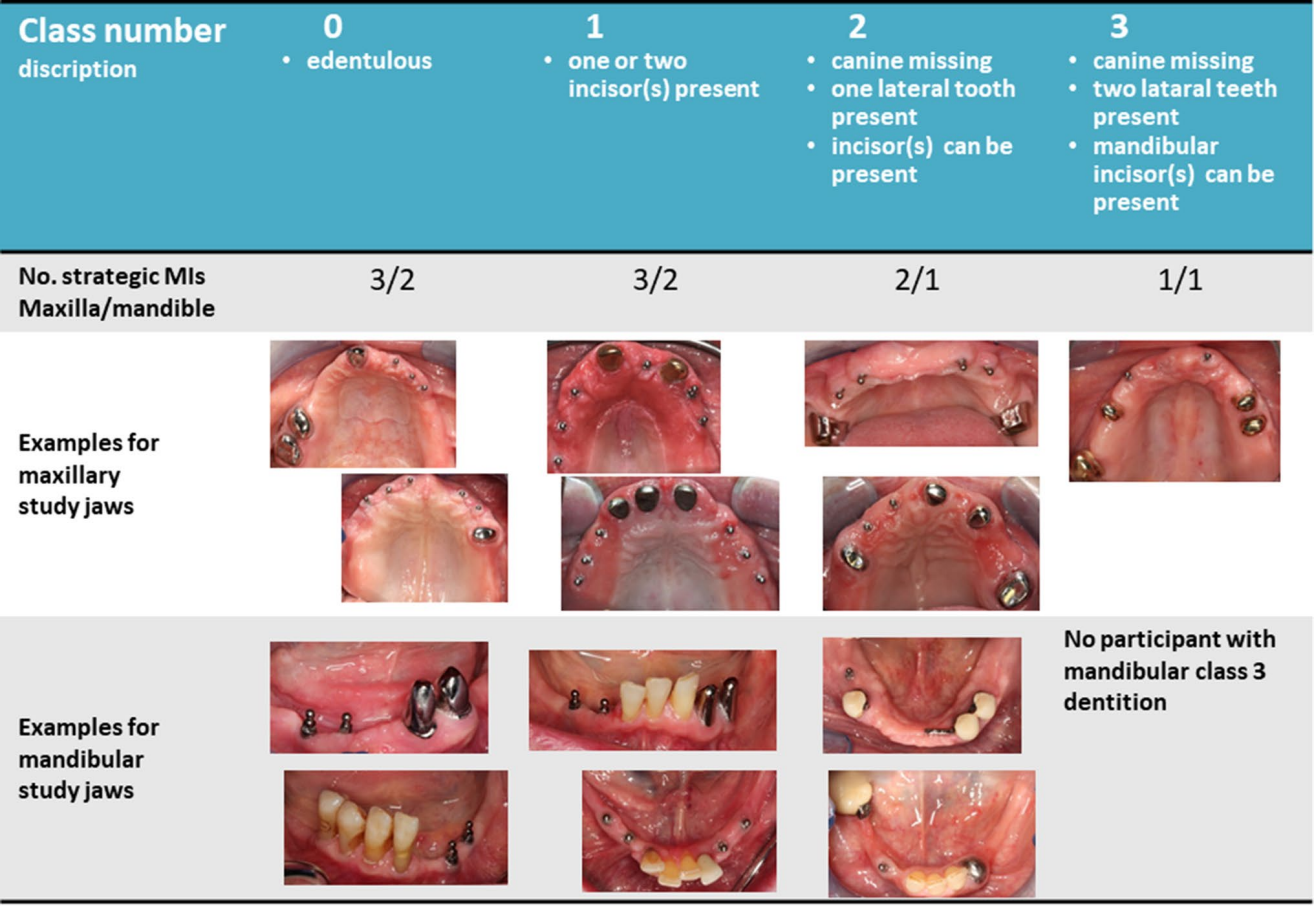
Classification on quadrant level per study jaw and clinical examples including placed strategic mini-implants

**Fig. 2:**
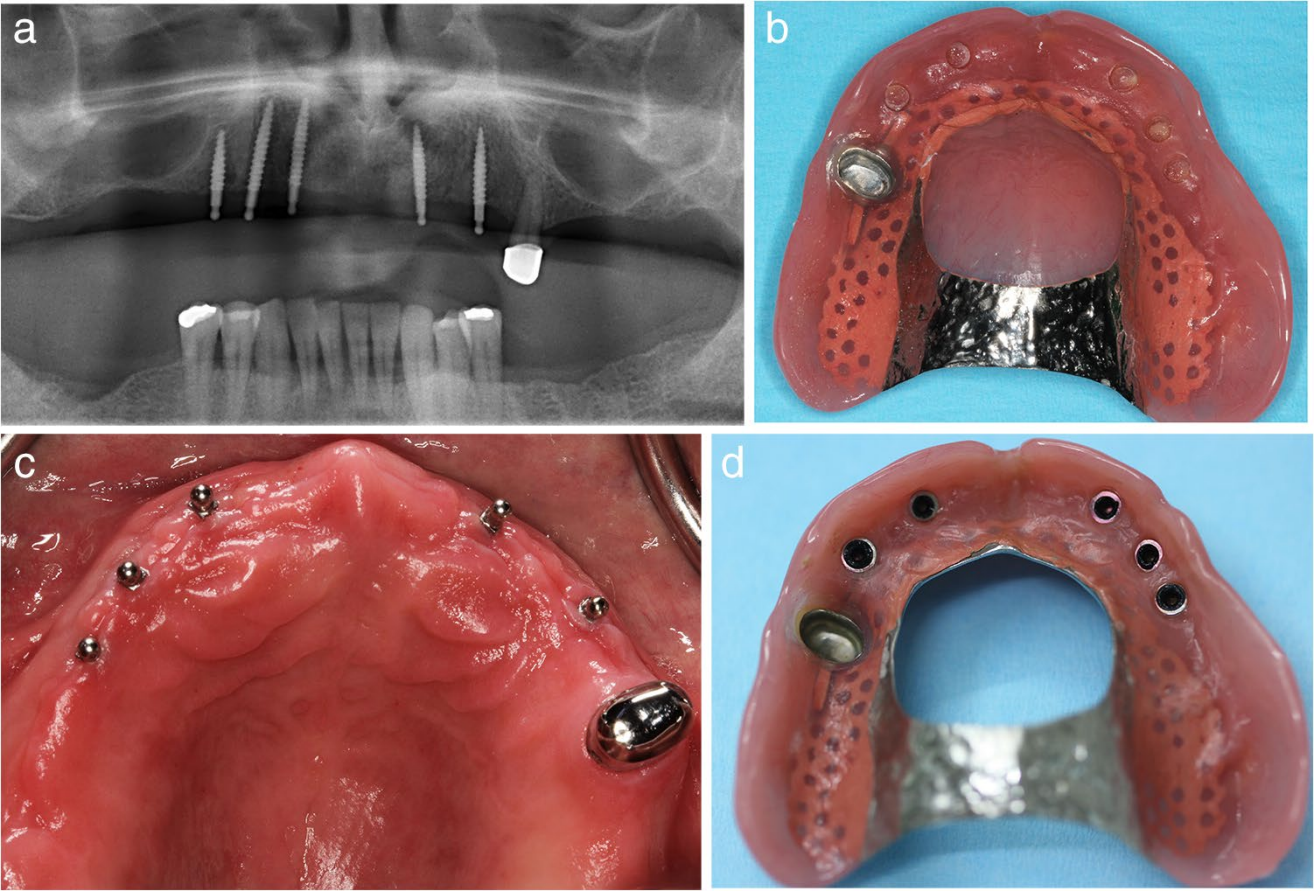
Mini-implants for the additional support of the double crown-retained removable partial denture in the maxilla remained initially unloaded in group B. After 4 months the housings were picked-up, and the palatal base was reduced

This multicenter RCT in a university hospital and three dental practices was approved by the Ethics Committee of the Greifswald University (BB 058/13A). All participants gave their written informed consent prior to inclusion in the study. Participants were patients with an RPD in one or both jaws with comparably unfavorable tooth distributions, i.e., one quadrant was edentulous (class 0), or one/both quadrants had either only anterior teeth (class 1), one (class 2), or at most two posterior teeth (class 3) and no canine (Fig. 1). The quadrant with the lowest class number determines the classification of the study jaw. The sample size calculation is based on bone level changes as the primary outcome and resulted in 26 participants per group. One-piece MIs (Mini Dental Implant, MDI, Manufacturer in the past 3 M ESPE and now Condent, Germany) with diameters of 1.8, 2.1, and 2.4 mm; lengths of 10, 13, and 15 mm; and ball attachments were inserted according to the quality and quantity of the available jaw bone. The participants received as many MIs from experienced dentists until either two abutments per quadrant (teeth plus MI) in the mandible or three abutments per quadrant in the maxilla were in place (Figs. 1 and 2). The higher maxillary abutment number corresponds with recommendations of researchers, clinicians, and manufacturers for MIs in the edentulous jaws [14].

The RPD was hollowed out over the ball abutments. Thereafter, the participants were allocated to the immediate loading group A or the delayed loading group B according to the 1:1 randomization ratio stratified by jaw and center. A sealed opaque envelope with the randomization detail was usually opened by the treatment coordinator. The surgeon was informed per telephone about the group allocation after implant placement. According to previous good experiences [15] and to the manufacturer guideline, immediate loading with matrices (O-ring housings) was only performed provided ≥ 35 Ncm insertion torque of all MIs. Alternatively, the RPD was soft relined, embracing the MI balls. In group B, the MIs were kept without loading for 4 months. Subsequently, the O-ring housings were picked-up in both group B and the soft relined RPDs in group A (Fig. 2).

### Data assessment

An independent expert dentist examined the patients before the surgery (*T*_−1_), after 2 weeks (*T*_1_), 4 months (*T*_2_), 4.5 months (*T*_3,_ after housing pick-up for the patients with soft relined RPDs of group A and all patients of group B), 1 year (*T*_4_), 2 years (*T*_5_), and 3 years (*T*_6_). Additionally, the stability values just after MI placement (*T*_0_) were measured in the university hospital since there was only one trained examiner with the calibrated devices attended at the time of surgery.

Implants that soundly maintained their function were considered to have survived. Removals or spontaneous losses of implants were defined as failure [33].

**Fig. 3:**
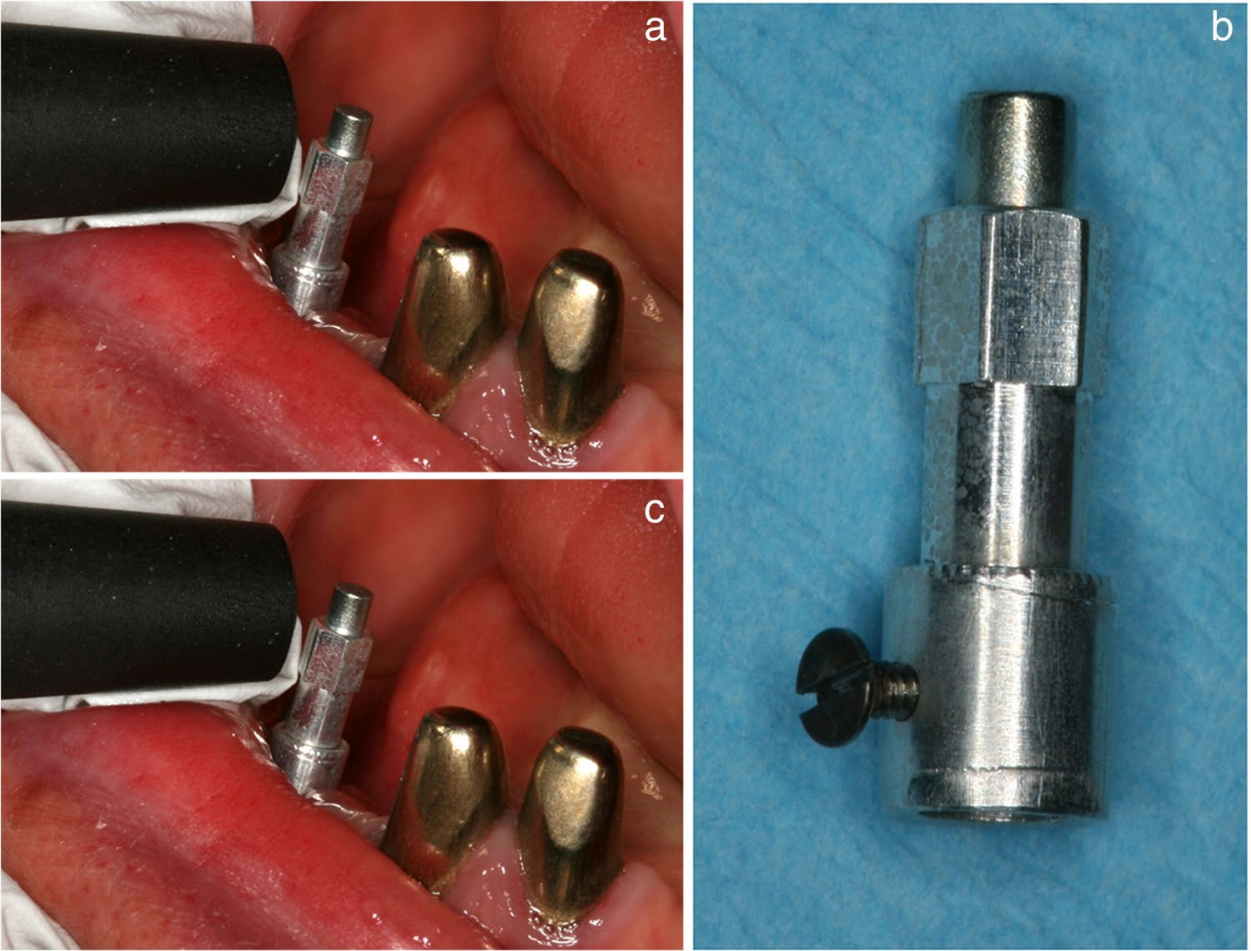
Stability measurements at mini-implant using the Periotest device (top) and the resonance frequency analysis (bottom). SmartPeg prototype (right) was put on the mini-implant and connected using a lateral screw

The Periotest instrument (Medizintechnik Gulden, Bensheim, Germany) was used to measure MI stability [34, 35]. A percussion rod impacts at right angles to the middle of the MI ball attachment 16 times for 4 s (Fig. 3).

The more stable the bone anchorage, the quicker the percussion is. The instrument measures the time that the percussion rod is in contact, with a shorter contact time indicating more stability. The computer converts the information to the Periotest value (PTV) on a scale between − 8 and + 50. PTVs below 0 are intended to be indicative for osseointegrated conventional implants [34].

Resonance frequency analysis (RFA) with the Osstell device (Osstell, Gothenburg, Sweden) was additionally used to measure MI stability [19, 34, 35]. The former manufacturer of the MI system for this study developed a SmartPeg prototype. The SmartPeg was put on the MI and fixed below the ball equator with a lateral screw (Fig. 3). The probe of a handpiece stimulates the SmartPeg that produces lateral stress in increasing frequency until the implant vibrates. The resonance was recorded and displayed by the measuring device. The implant stability quotient (ISQ) indicates the resonance frequency on a scale of 1–100. ISQ and implant stability are positively correlated. Most of the studies found mean RFA values of > 60 for conventional implants at surgery followed by a slight increase over time [19].

### Statistical analyses

Continuous variables are represented as median (1st–3rd quartile) because of asymmetric data distributions. To compare stability values between the treatment groups, mixed models were used on three levels, namely person, tooth site, and time as continuous variable [36]. The interaction between treatment and time was adjusted for sex, age, jaw, tooth site, and center [37]. Deviations from linearity for “time” were modeled by restricted cubic splines with three knots [38]. The tolerance limits of non-inferiority of immediately loaded MI versus delayed loaded MI were set at + 1 unit PTV and at − 2.5 units ISQ.

The Kaplan–Meier analyses were performed as descriptive statistics assuming independent observations. However, the Cox regression considered dependence among implants from the same patient by robust variances [33]. Defined *α*-levels were not regarded, and 95% CIs are primary presented to follow the recommendations of the American Statistical Association [39]. All statistical analyses were performed using Stata (Stata, Version 16.1; Stata Corporation, College Station, TX, USA).

## Results

### Participant’s characteristics

**Fig. 4:**
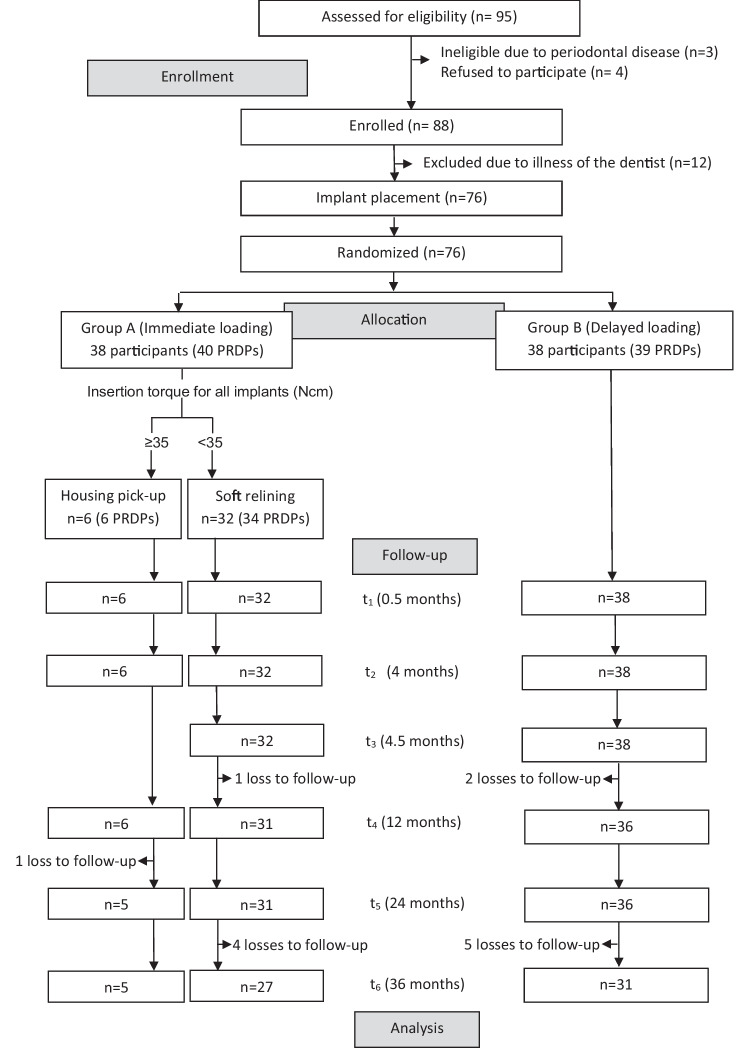
Flowchart of the participants

Because one of the participating surgeons had a long-term illness, 12 participants received no MI in the study period (Fig. 4).

**Fig. 5:**
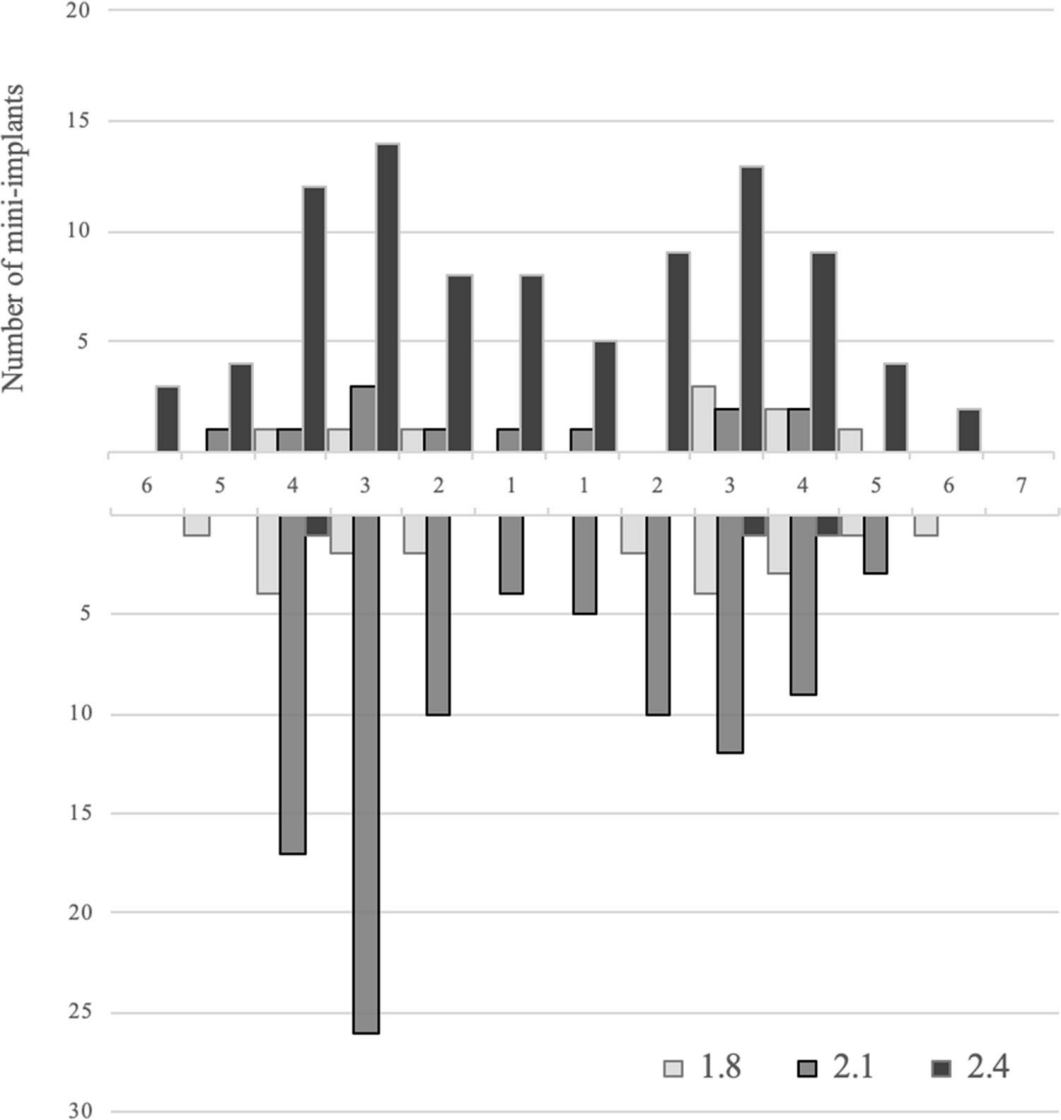
Distribution of mini-implants by tooth site (FDI tooth numbering system) and screw diameter

The recruitment period ranged between January 2014 and July 2015. The study ended in July 2018. The new or optimized RPDs of the 79 study jaws (31 maxillae) were either double crown-retained (*n* = 52), retained by double crowns and clasps (*n* = 15), clasp-retained (*n* = 10), or precision attachment-retained (*n* = 2) which were worn for at least 2 months. The median of the residual teeth was two in the maxilla (1st quartile = 2; 3rd quartile = 4) and three in the mandible (1st quartile = 2; 3rd quartile = 5). Participants with study maxillae showed on the opposing jaw: RPDs (*n* = 19), fixed dental prostheses (*n* = 9), or natural dentition (*n* = 3). Participants with study mandibles showed on the opposing jaw: complete dentures (*n* = 10), RPDs (*n* = 30), or fixed dental prostheses (*n* = 8). A total of 112 maxillary (median 3 per jaw) and 120 mandibular (median 2 per jaw) MIs were placed. Three participants received MIs in the maxilla and mandible. As seen in the flow chart (Fig. 4), each 38 participants were allocated to groups A (mean age 66.4 years, 22 women) and B (mean age 65.4 years, 25 women). In group A, 12 maxillary and 22 mandibular RPDs were primarily soft relined because the insertion torque of at least one MI was < 35 Ncm. The MIs of three maxillae and three mandibles were immediately loaded with housings (insertion torque ≥ 35 Ncm). The MIs of 16 maxillae and 23 mandibles in group B were delayed loaded. A total of six participants in group A (16%) and seven participants in group B (18%) were lost to follow-up in the whole study period, among them each two per group until the second year. Figure 5 shows the distribution by tooth site and MI diameter. In the maxilla, MIs with a diameter of 2.4 mm were predominantly placed, whereas in the mandible, the diameter of 2.1 mm and 1.8 mm predominated.

### Mini-implants stability

The median of maximum insertion torque of 222 MI was 25 Ncm (1st quartile, 20 Ncm; 3rd quartile, 35 Ncm). The different number of stability measurements and the higher number of PTVs compared with RFA values (total number 1270 versus 897), especially after the first year, has several reasons (Table 1).

**Table 1:** Median and quartiles for all mini-implant stability measurements at insertion and follow-ups

		Periotest				Resonance frequency analysis			
Time point		*N*^a^	1^th^ Q^b^	Median	3^rd^ Q	*N*	1^st^ Q	Median	3^rd^ Q
Insertion	*t*_0_	72	0.6	1.6	3.6	77	41	44	49
2 weeks	*t*_1_	222	1.7	3.6	6.3	217	32	39	43
4 months	*t*_2_	205	2.8	4.5	7.8	203	30	37	43
4.5 months	*t*_3_	193	2.6	4.5	7.3	195	32	39	43
1 year	*t*_4_	209	2.0	3.5	6.2	161	35	41	44
2 years	*t*_5_	196	2.0	3.5	6.6	36	39	43	46
3 years	*t*_6_	173	1.8	3.4	5.6				
Total		1270	2.0	3.6	6.6	889	32	39	44

^a^Number of measurements.

^b^Quartile.

First, the response rate for visits of the participants varied between follow-up points. Second, just after surgery (*T*_0_), stability values were available in the university hospital exclusively. Third, if the mucosa covered the MI insertion square, a safe fixation of the SmartPeg and subsequent measurement was sometimes impossible. Fourth, during the second year of the study, the manufacturer transferred the fabrication of the MI system to another company, and supplies of SmartPegs were stopped. Therefore, 3-year RFA measurements were not conducted contrary to the original study protocol. According to Table 1, primary PTV and RFA values from the university hospital suggest the highest MI stability at the day of placement.

**Fig. 6:**
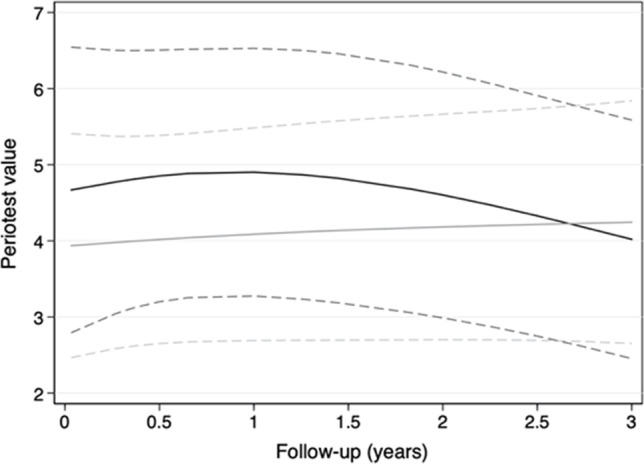
Adjusted Periotest values and the 95% confidence intervals (dashed line) of group A (black lines) and group B (gray lines) over the 3-year study period

**Fig. 7:**
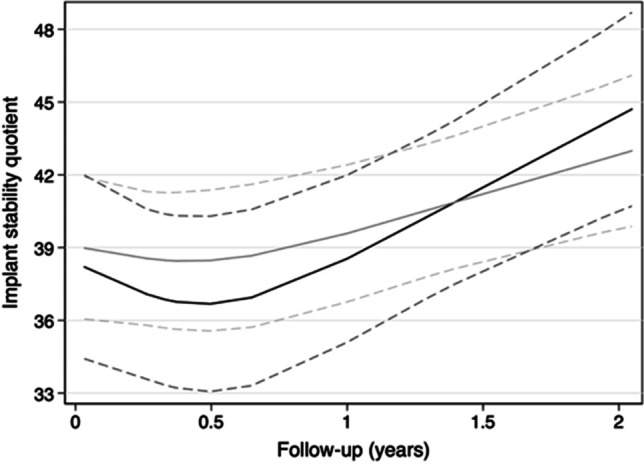
Adjusted values of the resonance frequency analysis and the 95% confidence intervals (dashed line) of group A (black line) and group B (gray line) over 2 years

The adjusted values and 95% CIs of the follow-ups by groups in Figs. 6 and 7 correspond with women, age of 65 years, mandible, first premolar site, and first study center. Assuming a tolerance limit of + 1 for PTVs, the immediately loaded MI of group A showed no inferiority compared with delayed loaded MIs of group B (Fig. 6), and the differences between groups are statistically not significant (*P* = 0.788). The 95% CI after 3 years is compatible with effects between − 1.4 (in behalf of group B) and 1.9 (in behalf of A). Similar results were obtained for the RFA values (Fig. 6). The 95% CI after 2 years is compatible with effects between − 5.2 (in behalf of group B) and 2.0 (in behalf of A) without a statistically significant difference (*P* = 0.390). However, in the first year of observation, PTVs tended to be higher, and RFA values tended to be lower with wide overlapping 95% CIs for the immediately loaded MIs of group A compared to group B. The values of the groups converged after the first year.

**Fig. 8:**
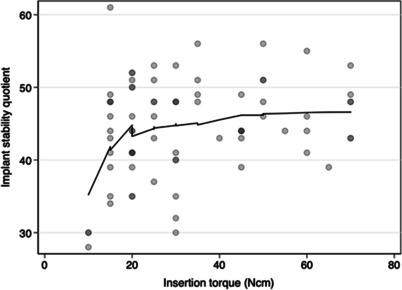
Scatter plot illustrating the relationship between insertion torque and resonance frequency analysis

The Spearman correlation of *r*_*s*_ =  − 0.83 between PTV und RFA values (*n* = 887) suggests a high negative correlation on condition of independent observations. The negative correlation of the maximum insertion torque with PTVs was weak (*r*_*s*_ =  − 0.15), whereas the positive correlation with RFA values was moderate (*r*_*s*_ = 0.24) as seen in Fig. 8.

**Fig. 9:**
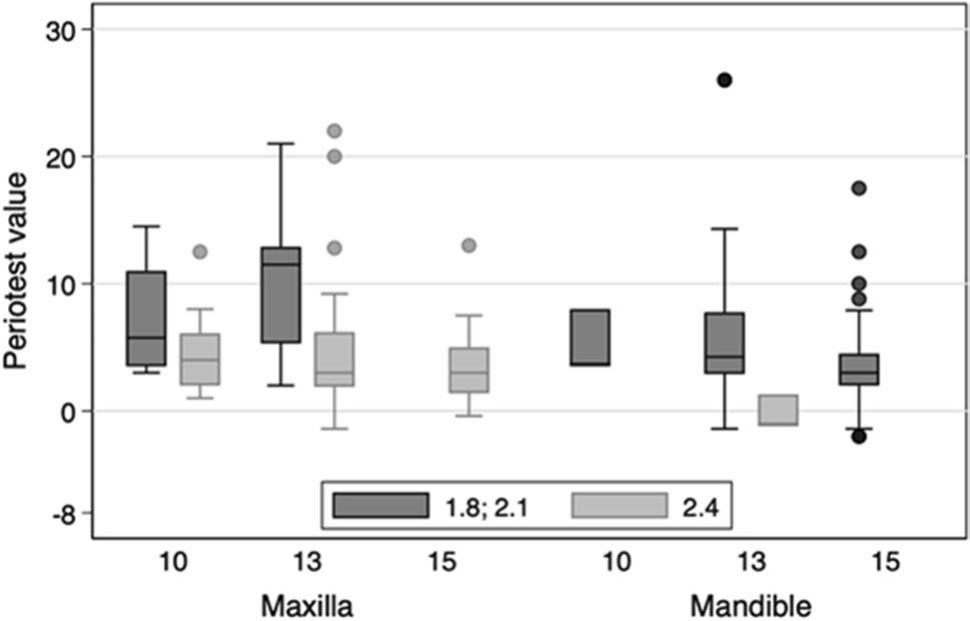
Periotest values by implant diameter and jaw at the 1-year follow-up

Figure 9 shows the PTVs by MI diameter and length in the maxilla and mandible at the 1-year follow-up. The diameters of 1.8 and 2.1 mm are pooled because of the low number of the thinnest MIs. The stability of 2.4 mm MIs was higher (mean PTV for all lengths together: 4.1 in the maxilla and − 0.3 in the mandible) than the stability of MIs with lower diameters (mean PTV for all lengths together: 9.8 in the maxilla and 4.7 in the mandible). The differences between MI lengths were negligible.

### Mini-implants survival rate

**Fig. 10:**
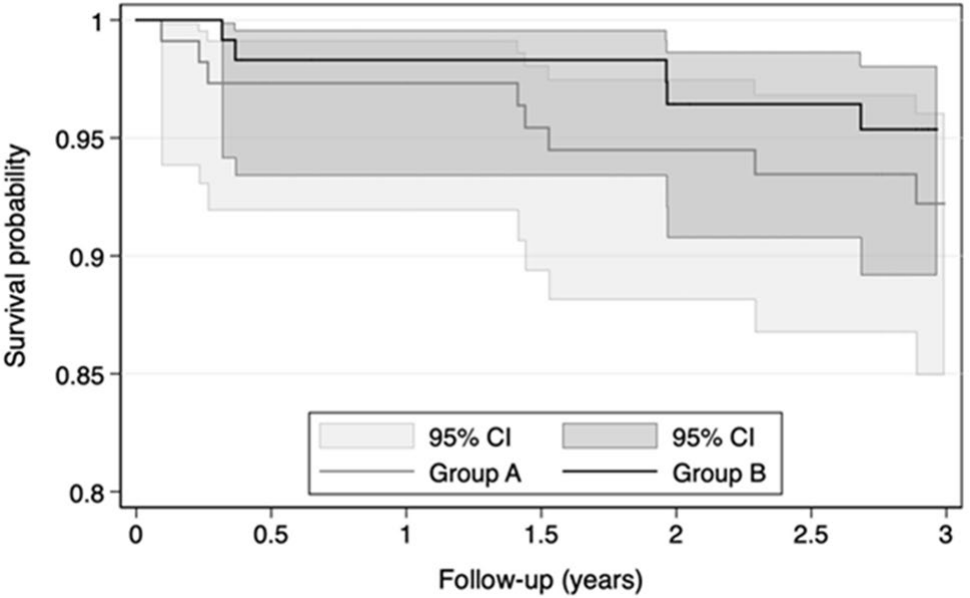
Survival rate probabilities of mini-implants by group

**Fig. 11:**
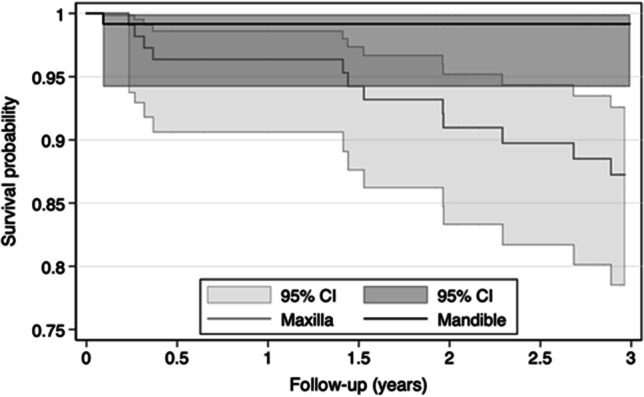
Survival rate probabilities of mini-implants by jaw

A total of 8 failures were registered in three participants of group A versus 5 failures in two participants of group B, which resulted in cumulative 3-year survival rates of 92% versus 95% (Fig. 10). In group A, one maxillary MI was misplaced and could not be covered by the RPD because of its buccal malposition. This MI was immediately removed. All other MI failed due to lost osseointegration. Each three losses in three maxillae, two losses in one other maxilla, the malpositioned MI, and another loss in one mandible yield MI survival rates of 87% for maxillary and 99% for mandibular MIs (Fig. 11).

According to Cox regression analyses considering dependent observations within one person, the 95% CI for the difference between the groups was 0.1 to 3.4 (hazard ratio [HR] = 0.6, *P* = 0.554). The 95% CI for the difference between the jaws was 1.6–142.2 (HR = 15.3; *P* = 0.017). Participants with MI losses wore either RPDs with multiple abutments (*n* = 3) or had natural dentitions (*n* = 2) in the opposing jaw. One patient with three MI losses was a current heavy smoker, and the other patients with MI losses were never smokers. The lost mandibular MI was placed in a fresh extraction socket contrary to all other MIs. MI failures were either spontaneous losses during removing the RPD at home (*n* = 6) or MI loosenings (*n* = 6) with subsequent removal without any severe inflammatory reaction of the peri-implant tissue or pronounced bone defects.

### Periotest values and implant survival

**Fig. 12:**
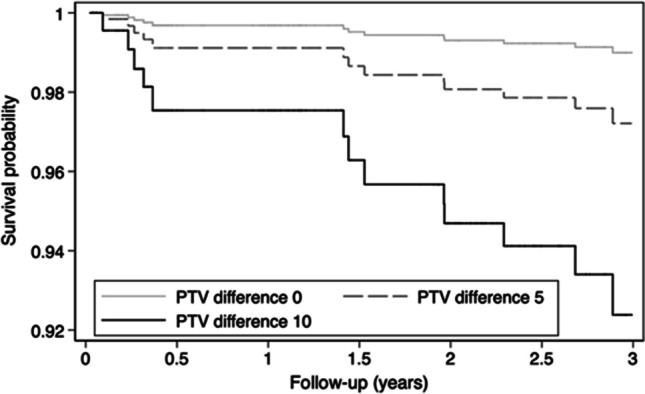
Survival rate probabilities by Periotest value differences according to Cox regression analyses

The majority of PTVs for failed MIs (8 out of 12) showed an increase between 2 weeks after placement (*T*_1_) and the follow-up before the loss (Table 2). According to Cox regression analyses, plus one unit PTV increases the failure risk (HR = 1.23; 95% CI = 1.15–1.31; *P* < 0.001). An increase of 5, 10, and 15 units of PTV results in HRs of 1.8, 7.9, and 22.0. Transformed in curves, the 3-year survival rate probability decreased from 97% in a PTV difference of 5 to 92% in a difference of 10 units (Fig. 12).

**Table 2:** Periotest values of failed mini-implants and the duration up to loss

No^a^	Group	FDI^b^ tooth site	Periotest values at					Loss after
			2 weeks	4 Mo^c^	4.5 Mo	1 yr^d^	2 yrs	
1	B	22	0.3	27.5				4 Mo
		23	1	4.6	6.3	13	24	32 Mo
2	A	13	− 0.8	3.1	4.4	9.2		18 Mo
		23	1.9	25				4 Mo
		24	5.9	10.7	12.5	19		17 Mo
3	A	21	2.5	5.4	5	3.6	6.4	35 Mo
		22	3.8	6	7.2	9.2	28	28 Mo
		23	5.5	24.5	23	22		17 Mo
4	A	42	1					1 Mo
5	B	11	32	28	32			5 Mo
		12	12.5	11	-	8.2		24 Mo
		13	5.3	7.3	-	12.5		24 Mo

^a^Participant number with mini-implant loss.

^b^Federation Dentaire Internationale tooth numbering system.

^c^Months.

^d^Year.

## Discussion

The primary outcome of this randomized trial was bone level changes of either immediately loaded MI in group A or delayed loaded MI in group B as supplementary abutments under existing RPDs. The assessment of bone heights at MIs on the panoramic radiographs are more sophisticated than expected, and the analyses are still pending. Thus, the present findings are descriptive and explorative in nature and not based on formal statistical hypothesis testing. The outcomes suggest no relevant differences between immediate and delayed loading regarding the survival rate and the stability of strategic MIs contrary to our hypotheses. The median of MI PTVs was higher, and the median of RFA values was lower than the stability values of standard-diameter implants whereby MI PTVs and RFA values strongly correlated. The correlation between insertion torque of MIs and their measured primary stability was rather moderate. The 3-year survival rate of mandibular MIs was markedly higher than of maxillary MIs (99% versus 87%, respectively). The expected relationship between increasing PTVs and the risk of implant loss was confirmed.

Some limitations of the trial besides participant`s response merit consideration. First, both upper and lower jaws with various tooth distributions were included. However, it was given that the few remaining teeth provided insufficient and/or imbalanced support for the RPD. Second, the different loading procedures dependent on insertion torque of MIs in group A limit a sound comparison with group B. We would reduce the risk of implant failures in the maxilla as seen in studies in which all maxillary MIs were immediately loaded with the housings [40, 41]. Third, the selection of the respective MI for every individual site depended on the bone height regarding the length. The quality and thickness of the bone determined the MI diameter as in clinical routine. Therefore, the thickest MIs with wide thread flanks were more frequently used in the spongier maxilla and thinner MIs predominated in the more cortical mandible according to the manufacturer instructions and to a number of studies [13, 14]. Thus, MI stability comparisons between maxillae and mandibles are less conclusive. Furthermore, different treatment strategies for maxilla and mandible were evaluated by using survival estimations since the MI number and their dimensions varied by jaw. However, the loading conditions of the MIs between patients should be comparable since the total number and distribution of RPD abutments were similar after MI placement. Fourth, our SmartPeg prototype was not validated, and after the second year, new SmartPegs were no longer available. Therefore, we dispensed with 3-year RFA measurements altogether.

Overall, this is the first estimation of mid-term survival rate and stability data for strategic MIs stabilizing RPDs with unfavorable distribution of natural abutments. Because of the multicenter design (one university hospital and three private practices), a multifaceted patient cohort was treated by experienced dental practitioners and examined by one trained investigator who was not involved in the treatment. The MIs were placed without knowing the loading modus up to the participant’s allocation. For MI stability assessments, two different methods were used whose values strongly negatively correlated.

As hypothesized in the design paper [30], there was a trend for lower stability of immediately loaded MIs compared to delayed loaded MIs in the first year of observation. This could be explained by the occlusal and lateral forces of the RPDs on the MI in the bone remodeling period [18]. As seen by overlapping 95% CIs, any difference between the groups could not be proven by the present data. After the first year, the values of group A conformed to group B. The slight increase of the RFA values in both groups and the decrease of the PTVs in group A correspond with prospective studies using conventional implants [19]. In observational studies using immediately loaded MI in edentulous mandibles, the PTVs remained either relatively constant [20] or increased in the first 4 months followed by a slight decrease [23].

The reference levels for the stability of conventional implants in a successful osseointegration are PTVs < 1 and RFA values > 60 [19, 35]. In the present study, the median PTV of 4.5 after 3 years and the median ISQ of 41.5 after 2 years indicate an insufficient stability for prosthetic loading. Because of their deflection during the measuring procedure, MIs may have other stability values than two-piece implants with diameters ≥ 3 mm. This conjecture is supported by the trend for a higher stability of the thicker MI in our trial and by two other studies with mandibular MIs of similar dimensions. The studies showed mean PTVs of 6 ± 6 [23] and 2.1 after 1 year [22]. Contrary results were published in two more prospective studies. The researchers reported on PTVs of − 1.4 after 6 months [21] or − 4.2 after 3 years [20]. Exact descriptions of the measurement procedure in the studies mentioned above are lacking. In an animal study, another SmartPeg prototype was developed for the same MI system used in the present study [42]. The MIs were inserted into the tibia/femur of rabbits. The median ISQs were 53.3 at insertion and 60.5 after 6 months. The higher values could be explained by the better bone quality in rabbit limbs and the more stable anchorage mechanism of the SmartPeg embracing the MI insertion square. In a randomized clinical trial, the researchers compared two immediate loaded two-piece implants with a diameter of 3 mm referred to as MI with two immediate loaded conventional implants (diameter 3,75 mm) to retain 30 free-end RPDs in mandibular Kennedy Class I dentitions [29]. They found identical 1-year survival rates of 93.3% and similar mean ISQ (70 versus 77.6, respectively). However, 3 mm diameter implants should be rather assigned to diameter-reduced or narrow conventional implants [13, 14].

The possible association between implant diameter and their stability was confirmed by our explorative evaluations of PTVs by MI diameter. The analyses were separated to jaw levels because of the possible differences in the bone quality between the maxilla and the mandible [18, 35]. Since 2.4 mm MIs were mainly inserted in the maxilla and the 2.1/1.8 mm MIs were mainly used in the mandible, the results would be otherwise biased. The difference of 0.3 and 0.6 mm and the differences in the screw threads design between the highest and the other MI diameters were most likely the reasons for the higher PTVs of the thinner MIs. This assumption is supported by a study comparing the PTVs of two other one-piece MI systems [22]. After 1 year, the MIs with diameters of 2.1 and 2.4 mm had a higher mean PTV (+ 2) than MIs with a diameter of 2.8 mm (− 1.6).

An expected trend of more MI failures in the immediate loading group A than in the delayed loading group B (8 versus 5 failures or 8% versus 5% 3-year failure rate) was not statistically verifiable. It should be noted that one MI in group A was removed before loading because of its malposition. Immediate loading of conventional implants with mandibular overdentures are poorly documented and result in more implant failures than delayed loading, whereas comparable data for the maxilla are missing [43]. In the only randomized 3-year comparison in 4-MI mandibular overdenture, immediate loading with housings had a lower MI survival rate than the delayed loading group (91.7% versus 96.7%, respectively) [44]. In the latter group, the MIs remained unloaded for 2 weeks, and thereafter, the overdentures were soft relined similar to our study. After 3 months, the housings were picked up.

The present MI survival rate of 99% in the mandible corresponds with results from strategic conventional implants (93–100%) [10, 11] and with the survival rate of MIs stabilizing complete mandibular dentures (92–100%) [12–14]. In a study, each two MIs with diameters of 2.0 or 2.5 mm were placed to retain 38 mandibular free-end RPDs. Before loading two MIs were lost, and the patients were excluded. This was classified as surgical survival rate of 97.4%. All loaded MIs survived at least 6 months [28].

Despite soft relining of RPDs in the case of insertion torque < 35 Ncm in group A and the no-load osseointegration in group B, eleven maxillary MIs were lost, and the survival rate probability was only 87% including the misplaced MI. All were multiple losses occurring in four patients and could be caused by the following reasons. First, two of the participants wore double crown-retained RPDs with multiple abutments in the mandible, and two participants had a natural mandibular dentition. A high occlusal load from the opposing jaw is a suspected cause of MI failures for some researchers [27, 29]. One participant with two MI losses was a heavy smoker. Smoking is a proven risk factor for implant complications [33]. The main reason for the high maxillary failure rate is probably the poorer bone quality with more trabecular and lesser cortical bone than in the mandible [19, 35, 45]. Last of all, one MI loss may cause further losses by compounding overload to the remaining abutments, similar to a domino effect.

A comparable failure rate of 82.3% in a 2-year prospective cohort study was reported. The study sample size was 31 patients with edentulous maxillae, each received 6 MIs. All overdentures were soft relined for 6 months before the housings were picked-up [26]. There were 32 failures in 16 patients, among them one patient with 5 and another with 6 failures. One patient was a heavy smoker with natural teeth in the mandible, and the other was a non-smoker with a mandibular implant overdenture. Seventeen MIs in ten patients were replaced, contrary to our trial in which some of the patients received MIs after the 3-year period. Other researchers reported unacceptable failure rates between 20 and 45% and sometimes clustered failures within one patient if all maxillary MIs under overdentures were immediately loaded with matrices [40, 41]. Overloading results in micro-movements of > 150 Ncm that can hinder secondary osseointegration [18].

Indeed, the predictive value of longitudinal RFA or Periotest measurements for implant failures is feasible, but the evidence is still weak and lacking for MIs [19, 34]. In the present study, the crude PTVs in eight of twelve lost MIs noticeably increased compared to the baseline values after 2 weeks, and the PTVs in six MIs were at least 20 just before the failure. The Cox regression analyses confirmed the suspected association with a 1.2- to 2.8-fold failure risk for a difference of 1–5 PTV units. According to systematic literature reviews, some but not all researchers found a relationship between falling RFA or increasing PTVs and conventional implants failure. However, single readings using any of the measurement techniques are of limited clinical relevance [19, 34].

## Conclusions

Measured stability of MIs as supplementary abutments under existing RPDs is lower than of conventional implants without any relevant differences between immediate and delayed loading. There was a strong negative correlation between RFA values and PTVs, whereas the correlation between insertion torque and both measures of the primary stability was rather weak to moderate. MIs of 2.4 mm diameter showed lower PTVs than those of 1.8 to 2.1 mm diameter. The differences in the survival rates between the loading groups were slight (immediate loading: 95% and delayed loading: 92%, respectively). However, the MI survival rate in the maxilla was lower than in the mandible (87% versus 99%, respectively) with clustered observations in the maxilla (11 losses in 4 participants). Increasing PTVs can predict failing MIs. More studies are required to verify the present explorative analyses.

## Data Availability

Data are available on request from the corresponding author.
